# Post-infection symptoms following two large waterborne outbreaks of *Cryptosporidium hominis* in Northern Sweden, 2010–2011

**DOI:** 10.1186/s12889-015-1871-6

**Published:** 2015-06-04

**Authors:** Moa Rehn, Anders Wallensten, Micael Widerström, Mikael Lilja, Maria Grunewald, Stephan Stenmark, Malin Kark, Johan Lindh

**Affiliations:** Public Health Agency of Sweden, Solna, Sweden; European Programme for Intervention Epidemiology Training (EPIET), European Centre for Disease Prevention and Control (ECDC), Stockholm, Sweden; Department of Medical Sciences, Infectious Diseases, Uppsala University, Uppsala, Sweden; Department of Clinical Microbiology, Unit of Communicable Disease Control and Prevention- Östersund, Umeå University, Umeå, Sweden; Department of Public health and Clinical Medicine, Unit of Clinical Research Center - Östersund, Umeå University, Umeå, Sweden; Department for Infectious Disease Control, Västerbotten County Council, Umeå, Sweden; Department of Cell and Molecular Biology, Microbiology, Uppsala University, Box 256, 751 05 Uppsala, Sweden

**Keywords:** Cryptosporidium hominis, Post-infection symptoms, Cryptosporidiosis, Sequelae

## Abstract

**Background:**

In 2010–2011, two large waterborne outbreaks caused by *Cryptosporidium hominis* affected two cities in Sweden, Östersund and Skellefteå. We investigated potential post-infection health consequences in people who had reported symptoms compatible with cryptosporidiosis during the outbreaks using questionnaires.

**Methods:**

We compared cases linked to these outbreaks with non-cases in terms of symptoms present up to eleven months after the initial infection. We examined if cases were more likely to report a list of symptoms at follow-up than non-cases, calculating odds ratios (OR) and 95 % confidence intervals (CI) obtained through logistic regression.

**Results:**

A total of 872 (310 cases) and 743 (149 cases) individuals responded to the follow-up questionnaires in Östersund and Skellefteå respectively. Outbreak cases were more likely to report diarrhea (Östersund OR: 3.3, CI: 2.0-5.3. Skellefteå OR: 3.6, CI: 2.0-6.6), watery diarrhea (Östersund OR: 3.4, CI: 1.9-6.3. Skellefteå OR: 2.8, CI: 1.5-5.1) abdominal pain (Östersund OR: 2.1, CI: 1.4-3.3, Skellefteå OR: 2.7, CI: 1.5-4.6) and joint pain (Östersund OR: 2.0, CI: 1.2-3.3, Skellefteå OR: 2.0, CI: 1.1-3.6) at follow-up compared to non-cases.

**Conclusions:**

Our findings suggest that gastrointestinal- and joint symptoms can persist several months after the initial infection with *Cryptosporidium* and should be regarded as a potential cause of unexplained symptoms in people who have suffered from the infection.

**Electronic supplementary material:**

The online version of this article (doi:10.1186/s12889-015-1871-6) contains supplementary material, which is available to authorized users.

## Background

*Cryptosporidium* is a protozoan parasite that can cause gastrointestinal illness in humans and animals [1, 2]. Several species of the parasite have been identified. *Cryptosporidium parvum* and *C. hominis* are the most prevalent species in humans. Cryptosporidiosis is transmitted mainly by the fecal-oral route, mostly by oocyst-contaminated water or food, or by direct contact with an infected person or animal [3].

Cryptosporidiosis occurs worldwide, and in all age groups [1], although children especially during the first year of life are frequently and severely affected [2, 4]. Numerous waterborne outbreaks have been reported globally [5] and the largest occurred in Milwaukee in 1993 where 400 000 people were infected through the public water supply [6].

In Sweden, *Cryptosporidium* has been a notifiable disease since 2004. Up to 2009 a mean of 45 (SD: 17) domestically acquired cases were reported annually [7]. However, the incidence of cryptosporidiosis is likely to be underestimated due to lack of testing [8, 9], especially in an outbreak situation where only a small proportion of probable cases are laboratory confirmed [10].

In otherwise healthy individuals infection with *Cryptosporidium* cause gastrointestinal illness. The most frequent symptoms are diarrhea, watery diarrhea, nausea, vomiting, fever and abdominal pain [1, 11] but infections may be asymptomatic [3]. Relapse of diarrhea more than two days after the end of initial diarrheal disease occurs in over a third of cases [10, 12]. Evidence of long-term gastrointestinal symptoms after initial infection is limited but persisting diarrhea up to three months after initial infection has been reported to be more frequent among cryptosporidiosis cases than controls [13]. Additionally, a recent follow-up study of 53 *Cryptosporidium* cases (without control population) indicates persisting gastrointestinal symptoms in two to eight patients up to three years after initial infection [14]. Similar to other gastrointestinal pathogens, *Cryptosporidium* infection may also cause reactive arthropathy. Reactive arthropathy after *Cryptosporidium* infection was first described in case reports in the 80s [15, 16]. A more recent prospective epidemiological study suggests presence of joint related symptoms up to three months after the original infection [13]. In immunocompromised patients the symptoms may be severe with a prolonged or chronic infection [1].

In 2010–2011, two large waterborne outbreaks caused by the same *Cryptosporidium* genotype (*C. hominis* IbA10G2) affected two cities in the Northern part of Sweden [10]. First in November 2010 in Östersund, a city of 60,000 people, and two months later in Skellefteå, a municipality of 72,000 people, situated approximately 450 km northeast of Östersund. Population-based cohort studies were performed in both cities, and estimated attack rates of 45 % in Östersund [10] and 28 % in Skellefteå (manuscript in preparation) were reported. Identified risk factors for *Cryptosporidium* infection in these outbreaks were high consumption of tap water in Östersund and living in a specific water supply region in Skellefteå. In order to better understand the consequences of these large outbreaks and to shed further light on whether *Cryptosporidium* infection may cause long term illness in those affected, we performed two separate follow-up studies using the outbreak cohorts in Östersund and Skellefteå.

## Methods

**Fig. 1:**
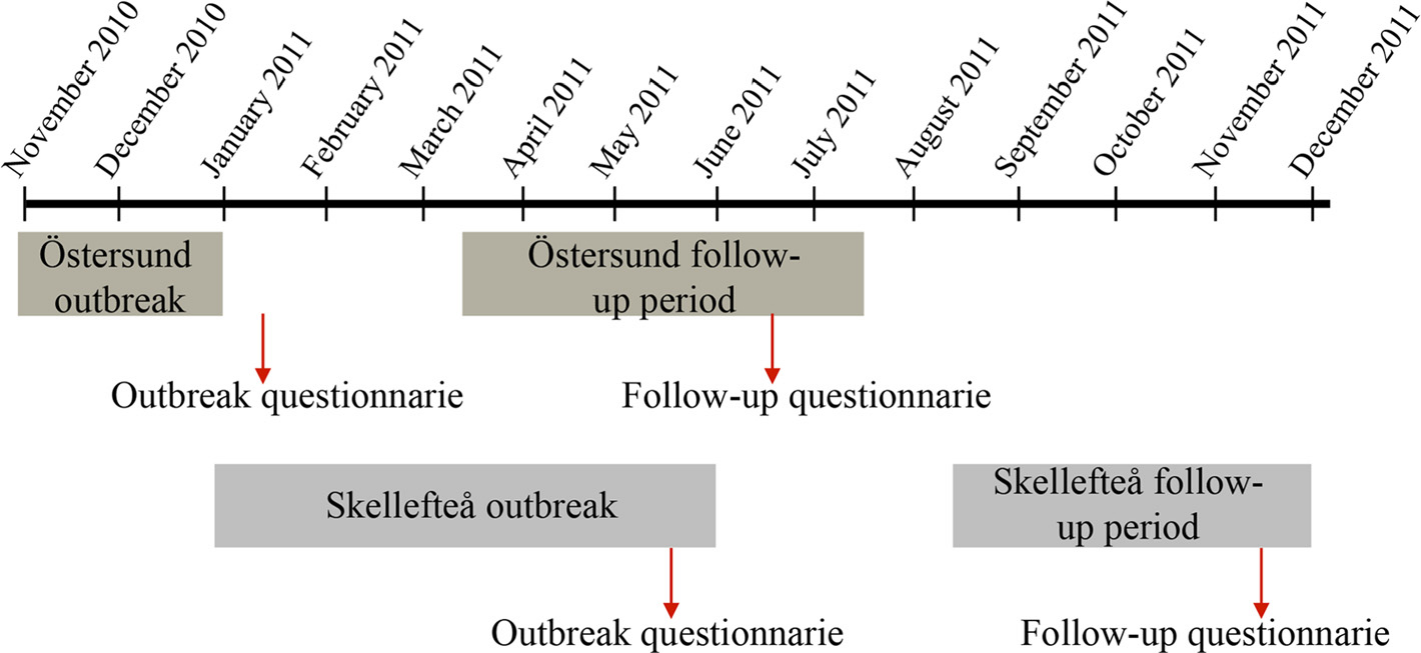
Graphic presentation of the timeline in the *Cryptosporidium* outbreak and follow-up studies in Östersund and Skellefteå, Sweden 2010–2011

For this present study, we used data collected in the outbreak cohort studies in order to determine case status (presence of symptoms during the outbreak, duration of symptoms), apply exclusion criteria (chronic diseases) and perform a non-response analysis. Each city’s follow-up study was analyzed separately. The outbreak and follow-up timelines is summarized in Fig. 1.

### The Östersund study

In mid-January 2011, two months after the onset of the outbreak, 1,524 people (2.5 %) in a random sample of persons living in Östersund were invited to take part in a retrospective cohort study to assess the magnitude of the outbreak, clinical characteristics and risk factors for disease [10]. The sample was randomly selected from the Swedish population registry by statistic Sweden (SCB). A total of 1,044 responded (69 %) to the outbreak questionnaire of which 481 were men (46 %). On 16 June, 2011, we invited these 1,044 respondents (henceforth referred to as the Östersund study) to complete a follow-up questionnaire regarding post-infection symptoms during the three months preceding the day of answering the questionnaire. Responses were recorded until 16 July, 2011, hence the period of recorded post-infection symptoms in the Östersund study was 16 March to 16 July, 2011. Parents or guardians were asked to respond for children <15 years of age.

### The Skellefteå study

In late May 2011, about five months after the onset of the outbreak, 1,750 people (2.4 %) in a random sample of the Skellefteå population were invited to take part in a retrospective cohort study in order to identify the start, magnitude and source of the outbreak, and risk factors for disease (manuscript in preparation). The sample was randomly selected from the Swedish population registry by statistic Sweden (SCB). A total of 1,099 persons responded (63 %) to the outbreak questionnaire, of which 532 were men (48 %). On 18 November, 2011, we invited these 1,099 respondents (henceforth referred to as the Skellefteå study) to complete a follow-up questionnaire regarding post-infectious symptoms after 20 August, 2011 until the day of answering the questionnaire. Responses were recorded until 15 December, 2011, hence the period of recorded post-infection symptoms in the Skellefteå study was 20 August to 15 December, 2011. Parents or guardians were asked to respond for children <15 years of age.

### Case definition

We determined outbreak case status in the Östersund and Skellefteå studies based on clinical characteristics reported in the outbreak questionnaire. We defined an outbreak case of *Cryptosporidium* infection as a respondent that reported new episodes of diarrhea (more than three loose stools in one day) and/or watery diarrhea and a duration of gastrointestinal symptoms of at least four days during the outbreak period in each city (Östersund: 1 November, 2010 to 31 January, 2011, and Skellefteå: 1 January, 2011 to 31 May, 2011). We defined respondents not fitting the case definition as non-cases during the outbreaks.

### Data collection

We sent postal questionnaires to the follow-up study population, accompanied with a letter explaining the study and a pre-paid envelope for return of the complete questionnaire to the Public Health Agency of Sweden. The Skellefteå study population was also given the option of completing the questionnaire online, accessed through a link that was sent in the accompanying letter. The follow-up questionnaire included questions about symptoms present during the follow-up period (as defined for each study above): loss of appetite, weight loss, diarrhea, watery diarrhea, bloody diarrhea, abdominal pain, headache, eye pain, stiff joints, fatigue, joint pain, swollen joints, joint discomfort, nauseas, vomiting, and a text area for any other symptoms. All returned paper questionnaires were optically scanned and transformed into an electronic database, and merged to the online questionnaire data (Skellefteå only). Demographic data were available from the population registry and were provided when the outbreak cohorts were established.

### Exclusion criteria

To avoid potential misclassification of case status, such as bias at follow-up linked to chronic gastrointestinal symptoms present for other reasons than *Cryptosporidium* infection, we excluded study participants that reported a diagnose of irritable bowel syndrome or inflammatory bowel disease in the outbreak questionnaire.

### Data analysis

We described the characteristics of the study populations in terms of symptoms present at follow-up, age and sex by outbreak case status. We examined symptoms at follow-up associated to outbreak case status by calculating odds ratios (OR) and 95 % confidence intervals (CI) obtained through logistic regression [17].

We excluded observations with missing values from the analysis. We included age and sex in the logistic regression models in order to adjust the association for their potential confounding effect. The analysis were performed in the statistical software R (R core team 2014) version 3.0.3 [18].

### Non-response analysis

We examined if sex, age, dwelling-place and case status of the study population were associated with non-response by calculating OR and 95 % CI using logistic regression modeling.

### Ethical statement

The study was reviewed and approved by the Stockholm regional research ethics review board (2011/220-31/4 and 2011/1289-32).

## Results

### Study population

A total of 872 and 730 individuals responded to the follow-up questionnaires in the Östersund and Skellefteå studies respectively (response rates 84 % and 68 %). In the Skellefteå study, 303 (44 %) responded to the questionnaire on-line. Before analysis we excluded 60 (41 non-cases and 19 cases) from the Östersund study and 44 (31 non-cases and 13 cases) from the Skellefteå study as they met the exclusion criteria. Finally, we included 812 individuals in the Östersund study and 686 in the Skellefteå study in the analysis. The characteristics of the study populations in each of the studies are presented in Additional file 1: Table S1. In the Östersund study 310 (38 %) were defined as cases in the outbreak of which 138 (45 %) were men. In the Skellefteå study, 149 (22 %) were defined as cases in the Skellefteå outbreak of which 73 (49 %) were men. The median age of cases was 32 years (range: 1–93) and 34 years (range: 2–92) in the Östersund and Skellefteå studies respectively.

### Symptoms during the follow-up period

Forty-nine percent of cases in Östersund and fifty-six percent of cases in Skellefteå reported symptoms at follow-up, most frequently fatigue, headache, abdominal pain and diarrhea (Table 1). The median number of symptoms among cases who reported symptoms present at follow-up was three in both Östersund (range: 1–10) and Skellefteå (range: 1–14).

**Table 1:** Symptoms reported at follow-up among respondents in the Östersund and Skellefteå studies, and its association with case status, Sweden 2011

	Östersund ^a^			Skellefteå ^b^		
Symptom	Cases n (%)	Non-cases n (%)	OR^c^ (95 % CI)	Cases n (%)	Non-cases n (%)	OR^c^ (95 % CI)
Weight loss	23 (7)	10 (2)	**4.0 (1.9–9.0)**	6 (4)	13 (2)	1.6 (0.5–4.2)
Watery diarrhea	36 (12)	18 (4)	**3.4 (1.9–6.3)**	22 (15)	27 (5)	**2.8 (1.5–5.1)**
Diarrhea	55 (18)	30 (6)	**3.3 (2.0–5.3)**	26 (17)	26 (5)	**3.6 (2.0–6.6)**
Abdominal pain	59 (19)	46 (9)	**2.1 (1.4–3.3)**	28 (19)	38 (7)	**2.7 (1.5–4.6)**
Rigid joints	37 (12)	38 (8)	**2.1 (1.2–3.5)**	13 (9)	32 (6)	1.5 (0.7–3.1)
Joint pain	34 (11)	39 (8)	**2.0 (1.2–3.3)**	21 (14)	44 (8)	**2.0 (1.1–3.6)**
Loss of appetite	48 (15)	42 (8)	**2.0 (1.3–3.1)**	18 (12)	37 (7)	1.5 (0.8–2.8)
Fatigue	80 (26)	78 (16)	**1.9 (1.3–2.8)**	40 (27)	75 (14)	**2.0 (1.2–3.2)**
Nauseas	44 (14)	40 (8)	**1.7 (1.1–2.7)**	27 (18)	53 (10)	**1.8 (1.1–3.1)**
Headache	74 (24)	77 (15)	**1.7 (1.2–2.5)**	39 (26)	97 (18)	1.4 (0.9–2.3)
Joint discomfort	28 (9)	35 (7)	1.7 (1.0–2.9)	-	-	-
Ocular pain	31 (10)	35 (7)	1.6 (1.0–2.7)	17 (11)	36 (7)	1.7 (0.9–3.1)
Swollen joints	12 (4)	18 (4)	1.5 (0.7–3.3)	7 (5)	21 (4)	1.2 (0.4–2.9)
Vomiting	20 (6)	34 (7)	0.9 (0.5–1.5)	10 (4)	25 (5)	1.1 (0.5–2.3)
Bloody diarrhea	2 (1)	2 (0)		3 (2)	0 (0)	-

^a^ 310 cases and 502 non-cases

^b^ 149 cases and 537 non-cases

^c^ Adjusted for age and sex

Bold indicates significant at 0.05 level

The results from the logistic regression models are presented in Table 1 and visualized in Additional file 2: Figure S1a and Additional file 3: Figure S1b. In both studies, outbreak cases were more likely to report diarrhea (Östersund OR: 3.3, 95 % CI: 2.0-5.3. Skellefteå OR: 3.6, 95 % CI: 2.0-6.6), watery diarrhea (Östersund OR: 3.4, 95 % CI: 1.9-6.3. Skellefteå OR: 2.8 95 % CI: 1.5-5.1) and abdominal pain (Östersund OR: 2.1, 95 % CI: 1.4-3.3, Skellefteå OR: 2.7, 95 % CI: 1.5-4.6) at follow-up compared to non-cases. Fatigue, nausea and joint pain was weakly, yet significantly, associated to being a case in the outbreak in both studies (Table 1). Further, weight loss, loss of appetite, stiff joints and headache was significantly associated with being a case in the outbreak in Östersund, but not in Skellefteå (Table 1).

### Non-response analysis

In both the Östersund and Skellefteå studies, the older age groups were more likely to respond to the follow-up questionnaire compared to the reference age group 0–5 years (Östersund 41–65 years OR: 1.7, 95 % CI: 1.1-2.5. Östersund >65 years OR: 3.4, 95 % CI: 1.9-6.2. Skellefteå >65 years OR: 3.4, 95 % CI: 2.2-6.4). Sex, dwelling-place and case status did not explain non-response in any of the studies.

## Discussion

We investigated post-infection health consequences following two separate waterborne *C. hominis* outbreaks in Northern Sweden based on data from a random sample of the population. The overall findings between the two studies are comparable and suggest that outbreak cases in both Östersund and Skellefteå were more likely to have gastrointestinal- and joint-related symptoms.

Case-based analysis of the time between initial infection and post-infection symptoms could not be performed in our studies because the individual time of completing the follow-up questionnaire was not documented. On study population level the time between initial infection and point of follow-up could vary between 2.5-8.5 months in Östersund, and 2.5-11.5 months in Skellefteå. Our findings suggest that the health consequences may persist even longer than stated in previous reports [13, 15] although persisting gastrointestinal symptoms between 24 and 36 months after infection have been described in up to eight of 53 *Cryptosporidium* cases [14]. Early childhood *Cryptosporidium* infection and diarrhea has been linked to impaired physical fitness 4–7 years later [19]. However this evidence comes from investigations in a low-income setting. Furthermore, similar long-term effects following initial infection have been recorded for other gastrointestinal parasites. For example giardiasis have been documented to cause long term gastrointestinal symptoms [20, 21] and fatigue [20–22] for several years following infection during a large waterborne outbreak in Norway.

The full consequences of the outbreaks that occurred in Östersund and Skellefteå cannot be evaluated through these studies. For example, we did not evaluate the severity and effect on sick leave among our study population. However the prevalence of health problems several months after the outbreaks suggests that total cryptosporidiosis-related cost of illness may be higher than what could be expected from only the infection.

Our results may be influenced by recall bias and non-response bias. Recall bias may affect cases and non-cases differently since cases may have been more self-observant due to the initial infection and therefore more prone to remember more symptoms. A proportion of the invited study population was lost to follow-up (16 % and 32 %). Non-response analysis did not suggest that cases were more likely to respond. Our findings derive from two waterborne outbreaks in Sweden with almost 50000 people estimated to be infected. Only a small proportion of the cases were tested and laboratory confirmed and no additional sampling was performed among our study population. The lack of laboratory confirmation is a limitation in our study and leads to four minor concerns regarding misclassification of case status:

First, during the outbreak periods, norovirus was circulating in Sweden and it is likely that some of our cases actually suffered from norovirus infection (or other gastrointestinal pathogens) rather than cryptosporidiosis. However, our case definition required a duration of symptoms of more than four days, which is longer than what is normally expected from norovirus infections [23], thus likely excluding potential norovirus cases. Additionally, *Cryptosporidium* was detected in the drinking water during the outbreak period in Östersund, suggesting that cases of gastroenteritis during this time were likely to be explained by *Cryptosporidium* infection [10]. The 149 laboratory confirmed cases of cryptosporidiosis related to the outbreak in Östersund were all negative for other gastrointestinal pathogens, including norovirus. During the peak in the Skellefteå outbreak (15–26 April, 2011), 45/74 laboratory confirmed *Cryptosporidium* cases were also analyzed for norovirus and one was positive. It is also likely that the frequency of misclassification of cases differs between the two studies due to the difference in duration of the outbreaks. The outbreak period in Skellefteå was estimated to have lasted for five months, a rather long time period when other pathogens could have caused similar symptoms in line with our case definition. In Östersund on the other hand, the outbreak was recognized and controlled within a month after it started [10], decreasing the time of risk for misclassification of case status. This may explain why a number of symptoms (weight loss, loss of appetite, stiff joints and headache) were associated with outbreak case status only in the Östersund study and why measures of association were often stronger in the Östersund study than in the Skellefteå study [10]. Furthermore, *Cryptosporidium* oocysts were identified in drinking water from Östersund [10] while no oocysts could be found in drinking water from Skellefteå (unpublished data). This suggests that the cases in Östersund consumed more oocysts then the cases in Skellefteå and therefor were more frequently and severally effected by infections and sequelae. However, these points remain speculative.

Second, persons who constantly suffer from intermittent diarrhea are likely to have been classified as cases in the outbreak and are also likely to report similar symptoms at follow-up. We made an attempt to minimize this effect by excluding cases with diagnosed irritable bowel syndrome or inflammatory bowel disease. Thus persons that have undiagnosed persisting gastrointestinal symptoms and reported them as new episodes of diarrhea may still be included in our studies and defined as outbreak cases as well as cases with symptoms at follow-up, resulting in an overestimation of the associations.

Third, if asymptomatic and mild cases suffered from *Cryptosporidium* related post-infection symptoms they are likely to contribute to the prevalence of symptoms in the non-case group at follow-up, thus diluting the true association, suggesting that our findings are underestimated.

Finally, we lack information about travel history during the outbreak- and follow-up periods, preventing exclusion of travel-related infections in our studies. The possible misclassification of outbreak cases due to non-cryptosporidiosis travel-related infections during the outbreak period may further dilute our results. Travel-related infections during the follow-up period are however a smaller problem since the travel related symptoms are likely to be evenly distributed among outbreak cases and non-cases.

## Conclusion

The data from these two studies conducted after two major *Cryptosporidium* outbreaks provide unique possibilities to describe post-infection health consequences. Our findings support previous reports that persisting gastrointestinal symptoms can continue several months after the initial infection with *Cryptosporidium*, suggesting that the public health consequences of the outbreaks that occurred in Northern Sweden go beyond the outbreak period and that the consequences of *Cryptosporidium* infections are underestimated worldwide.

### Implications

A history of cryptosporidiosis should be regarded as a potential cause in patients with long lasting gastrointestinal symptoms. Furthermore, our findings suggest that long-term symptoms need to be included in calculations on the total health and economic burden of cryptosporidiosis. Lastly, in order to find out how long-term symptoms may persist after infection, even longer follow-up periods of *Cryptosporidium* cases are needed.

## Additional files

Additional file 1: Table S1. Demographic characteristics of the Östersund and Skellefteå studies at follow-up presented by outbreak case status, Sweden 2011. Additional file 2: Figure S1a. Graphic presentation of associations between outbreak cases and symptoms at follow-up, Östersund, 2011. Additional file 3: Figure S1b. Graphic presentation of associations between outbreak cases and symptoms at follow-up, Skellefteå, 2011.
