# Supplementary material for: Post-infection symptoms following two large waterborne outbreaks of Cryptosporidium hominis in Northern Sweden, 2010–2011
Source: BMC Public Health. 2015 Jun 4;15:529. doi: 10.1186/s12889-015-1871-6 (PMC4454271; doi:10.1186/s12889-015-1871-6)
Supplement: Additional file 1: Table S1. — Demographic characteristics of the Östersund and Skellefteå studies at follow-up presented by outbreak case status, Sweden 2011. [file 12889_2015_1871_MOESM1_ESM.docx]

**Supplementary Table 1: Demographic characteristics of the Östersund and Skellefteå studies at follow-up presented by outbreak case status, Sweden 2011**

|  | **Östersund** | | | **Skellefteå** | | |
| --- | --- | --- | --- | --- | --- | --- |
| Characteristic | Cases n (%) | Non-cases n (%) | Total n (%) | Case n (%) | Non-cases n (%) | Total n (%) |
| *Sex* |  |  |  |  |  |  |
| Women | 172 (55) | 272 (54) | 444 (55) | 76 (51) | 273 (51) | 349 (51) |
| Men | 138 (45) | 230 (46) | 368 (45) | 73 (50) | 264 (49) | 337 (49) |
|  |  |  |  |  |  |  |
| *Age group (years)* |  |  |  |  |  |  |
| 0-5 | 89 (29) | 125 (25) | 214 (26) | 45 (30) | 110 (20) | 155 (23) |
| 6-15 | 46 (15) | 63 (13) | 109 (13) | 32 (21) | 120 (22) | 152 (22) |
| 16-40 | 58 (19) | 78 (16) | 136 (17) | 22 (15) | 30 (6) | 52 (8) |
| 41-65 | 90 (29) | 137 (27) | 227 (28) | 31 (21) | 113 (21) | 144 (21) |
| > 65 | 27 (9) | 99 (20) | 126 (16) | 19 (13) | 164 (31) | 183 (27) |
|  |  |  |  |  |  |  |
| Total | **310** | **502** | **812** | **149** | **537** | **686** |
