# Supplementary figures and images for: Post-infection symptoms following two large waterborne outbreaks of Cryptosporidium hominis in Northern Sweden, 2010–2011
Source: BMC Public Health. 2015 Jun 4;15:529. doi: 10.1186/s12889-015-1871-6 (PMC4454271; doi:10.1186/s12889-015-1871-6)

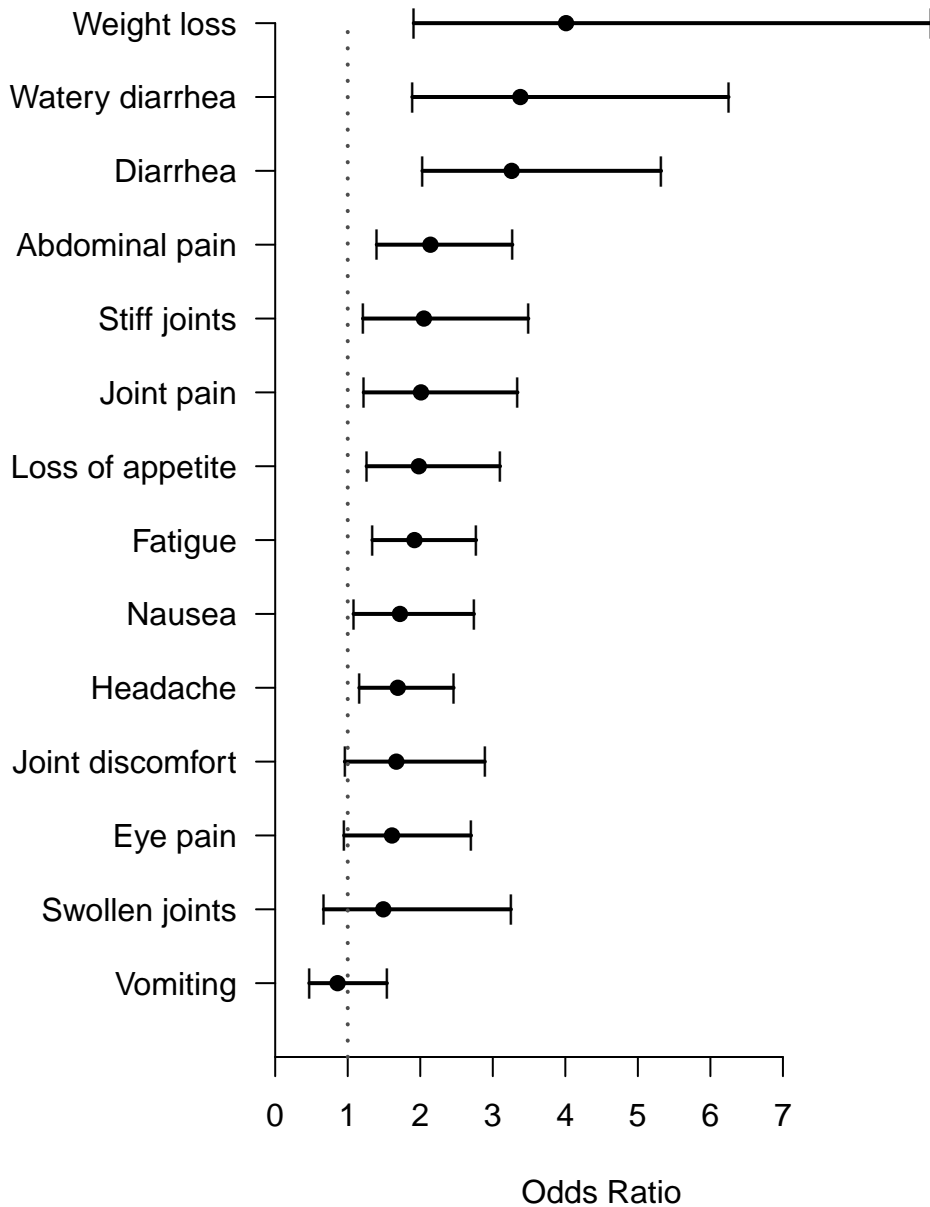

Supplement: Additional file 2: Figure S1a. — Graphic presentation of associations between outbreak cases and symptoms at follow-up, Östersund, 2011. [file 12889_2015_1871_MOESM2_ESM.pdf]

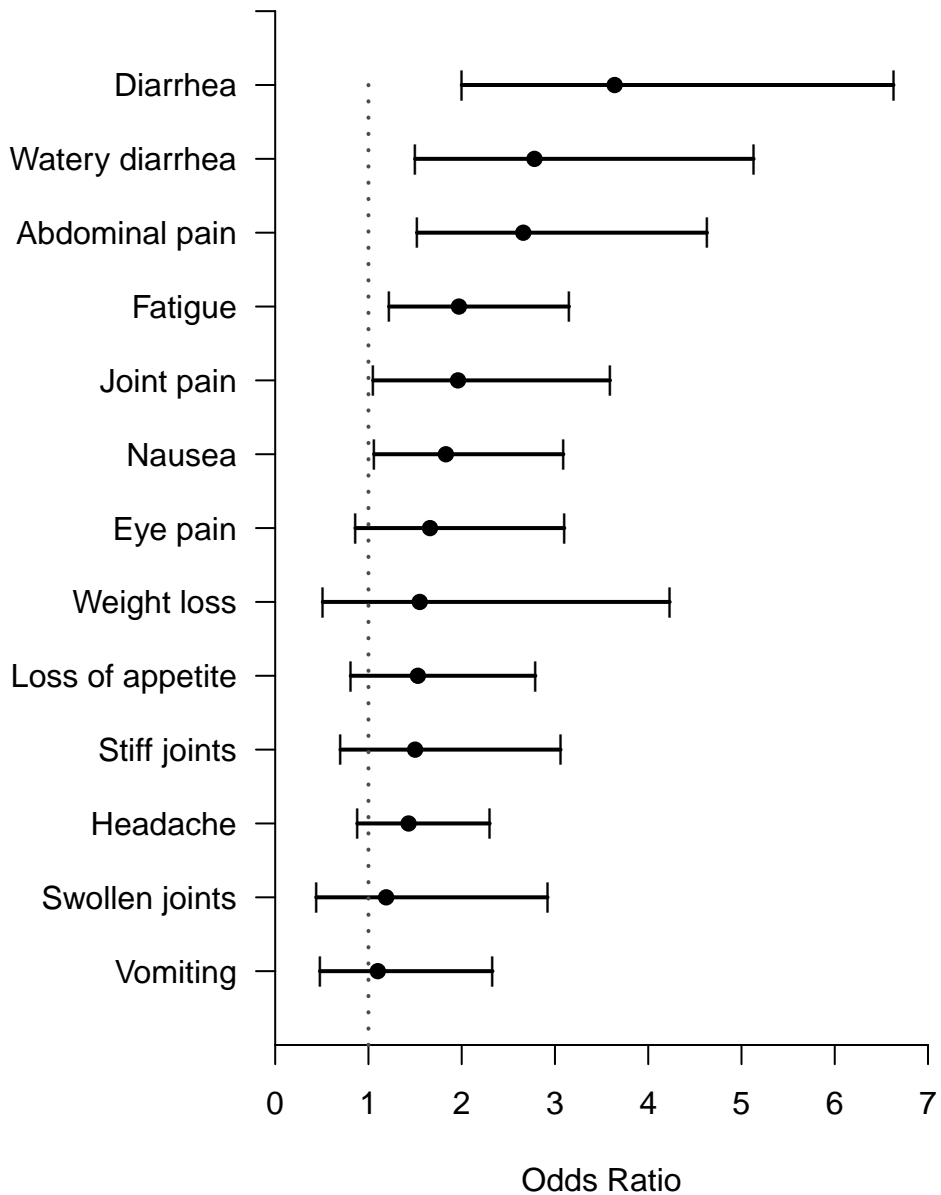

Supplement: Additional file 3: Figure S1b. — Graphic presentation of associations between outbreak cases and symptoms at follow-up, Skellefteå, 2011. [file 12889_2015_1871_MOESM3_ESM.pdf]
